# A New Duty of the Medical Profession: The Education of the People in Scientific Medicine1Presidential address delivered before the First District Branch of the Medical Society of the State of New York, at the second annual meeting held at Poughkeepsie, October 21, 1908.

**Published:** 1909-01

**Authors:** S. W. S. Toms

**Affiliations:** Nyack, N. Y.


### A New Duty of the Medical Profession: The Education of the People in Scientific Medicine.[1]

By S. W. S. TOMS, M. D., Nyack, N. Y.

IF I were to conform to the usual custom in acknowledging the honor conferred upon me on this occasion in presiding over this distinguished gathering of physicians, assembled from the six counties that comprise the First District Branch, I should be appropriating a distinction not meritoriously bestowed. "Some acquire greatness, others have greatness thrust upon them."

I was neither elected, selected, nor appointed to preside at this meeting as your president. I was commanded to do so by the secretary of the State Society in a letter received from him last March, because the gentleman, who by election and promotion should have filled the office had defaulted in the ranks; and because of this "It was up to me"—in the memorable words of our courteous secretary. If this imposes upon you any suffering, I assure you it is the fault of the by-laws in not providing a·second vice-president, upon whom I might shift any effect of your misfortune. I am here to fill a vacuum.

In my official visits to the county medical societies I have gained some impressions of society meetings and organisation, which seems to me an appropriate subject to present on this occasion. The papers and discussions on medical subjects, which it was my privilege to hear, were of a high order of merit, and marked evidence of harmony and good organisation,—the fruits of a reunited profession in this state,—were every where conspicuous.

There is a notable disposition by some societies for an organsied effort in protecting the public by suppressing quackery. New York county has been the pioneer and the best exemplar in this commendable warfare on the charlatans of all kinds. Crandall, in his report of seven years of official connection as a member of the Comitia Minora of the Medical Society of the County of New York, at its one hundred and second annual meeting, details in the most comprehensive manner the very successful work of that society. He very truly says: "Enforcement of medical practice laws, and the protection of the public against illegal and criminal practitioners, are among the duties which the county society owes to the profession and to the public." "The state and county societies in New York were organised pursuant to the important law of April 4, 1806, and the duty of

1. Presidential address delivered before the First District Branch of the Medical Society of the State of New York, at the second annual meeting held at Poughkeepsie, October 21, 1908.

regulating medical practice was placed on them by the State at their inception." Kings County also, I learn, has done admirable legal work in prosecuting quacks.

At a meeting, which I attended at White Plains, September 15, 1908, a report was presented of what had been accomplished by a letter written by the counsel for the state society, coöperating with the Medical Society of the County of Westchester, in suppressing a notorious illegal practitioner. The evidence had been secured by the medical society with proof sufficient to have convicted him, at a trifling expense, had he not left the city very promptly; and cases from Yonkers, New Rochelle and other places in the county have been likewise dealt with, with gratifying results.

The presidents of the county societies have appointed committees to secure data from all sections where illegal and itinerant practitioners are violating the medical act and imposing on the public. The state society is desirous of securing sufficient reliable information from such authentic sources as a basis for future legislation. The difficulty which confronts a medical society in prosecuting local imposters is very obvious. The district attorneys and magistrates owe their appointment to the suffrages of the locality, and are frequently disposed to regard complaints as persecutions; and it is for this very good reason the state society through its legal counsel can be a more effectual agent in suits to suppress this form of imposition on the public.

When a medical society undertakes to act for the public good, it deservedly commands respect and coöperation from public sentiment and esteem. A medical society should not exist for the mere intellectual and social self-interest of its members alone. There is a sphere of usefulness in impressing its influence on the public mind in various educational ways. The title of the presidential address of the American Medical Association, delivered at the fifty-ninth session: "A New Duty of the Medical Profession: The Education of the Public in Scientific Medicine," and more recently that of another address by Dr. M. Allen Starr, at the opening of the Medical Department of Columbia University: "The Duties of the Medical Profession to the Public," present convincingly such obligations.

Dr. Burrell, in his address outlines the objects of a medical society as follows: "First,—That individually its members may be better able to care for the sick. Second.—That they may collectively be better fitted to prevent disease. Third.—That they may know men and their ways and by social intercourse may live broader lives."

Besides the necessity for good organisation, harmony and individual enthusiasm for scientific progress, zeal for work is a prime factor. It is necessary that well defined plans should be conceived and assigned to appropriate committees in order to achieve that success which, at the expiration of each year, the president of each county society may, with pride, recount in his valedictory. The individual member in a county society becomes a unit in the state and national bodies, and upon him is conferred all the privileges these great medical associations possess. He is elevated into the house of peers of the profession. There is something wrong with the man who today is not an equal with his brothers in this respect.

The principles of these great and honored organisations are to uplift every member they include. Every county officer would do well to read the article by Osler, quoted by Dr. J. Riddle Goffe in his address delivered October 17, 1905, on "Organisation the Watchword of Creation." "In too many towns and smaller communities miserable factions prevail and bickerings and jealousies mar the dignity and usefulness of the profession. So far as my observation goes the fault lies with the older men. The young fellow. if handled aright and made to feel that he is welcomed and not regarded as an intruder to be shunned, is only too ready to hold out the hand of fellowship.

"The society comes in here as professional cement, The meetings in a friendly, social way lead to a free and open discussion of differences in a spirit that refuses to recognise differences of opinion on non-essentials of life as a cause of personal animosity or ill feeling. An attitude of mind habitually friendly, more particularly to the young man, even though you feel him to be the David to whom your kingdom may fall—a little of the old fashioned courtesy which makes a man shrink from wounding the feelings of a brother practitioner,—in honor preferring one another; with such a spirit abroad in the society and among its older men, there is no room for envy, hatred, malice or any uncharitableness. It is the confounded tales of patients that so often set us by the ears, but if a man makes it a rule never, under any circumstances to believe a story told by a patient to the detriment of a fellow practitioner, even if he knows it to be true, though the measure he meets may not be measured to him again, he will have the satisfaction of knowing that he has closed the ears of his soul to ninety-nine lies, and to have missed the hundredth truth will not hurt him. Most of the quarrels of doctors are about non-essentials, miserable trifles and annoyances—the pin-pricks of practice, which would sometimes try the patience of Job. But the good fellowship and friendly intercourse of the medical society should reduce these to minimums."

The short comings in the medical profession are by no means confined to "the many towns and smaller communities" of Dr. Osler's picture. The good feelings between doctors occasionally is marred by inconsiderate remarks at public clinics,—sometimes in the presence or hearing of the patients. It is not an uncommon thing for a doctor's patient to surreptitiously hie himself off to a clinic for various personal reasons, often with ulterior motives. They go without a card or letter of the attending doctor, and rarely intimate their intentions. They relate their case in prejudiced misstatement or evasions, often to the attending physician's detriment, and gloat over any criticism they chance to hear concerning their former treatment or diagnosis.

They frequently have been influenced by busybody friends and as the service they receive is free, they grossly abuse the privilege of charitable institutions. When these cases present themselves in this way no exceptions would be entertained if they were postponed to another clinic day, in the meantime the attending physician to be communicated with to ascertain all available facts concerning their cases. By such a course there could never exist any reason for hard feelings between the physicians in attendance and the clinician; on the contrary they would be enduring friends, avoiding an everlasting boycott of each other and mutually exchanging professional references in the future; at the same time disarming a patient wavering in his confidence in his doctor, who before wished to hear something that would possibly seriously injure his practice.

Medical societies should discourage the formation of medical cliques. It is not uncommon for a few men to band together for the purpose of controlling the society, or dominating the management of a local institution for ulterior or selfish motives. "Close communions" are well estimated in their purposes by the public sooner or later. It brings well merited reflection to those comprising them, but the incidental effects upon the institution or society with which they are officially associated are unfortunately such as to alienate the public support. These unprofessional methods smack loudly of the trust spirit of commercialism whereever they exist.

A languishing medical society should energetically investigate the causes for its conditions. A live thriving society is a most profitable and stimulating source of energy, of social and professional uplift to its members, but needs new leaven occasionally. A careful and searching diagnosis for its ills should be instituted and the cause removed. "Expectant treatment," is not appropriate in bringing back a healthful state. The means adopted should be combined vigorous, internal and external treatment as the illness may be in its terminal stages. The strict following of well conceived details, carefully outlined by the consultants, as the best course to follow in achieving the most promising results should be consistently adhered to with good nursing, the proper social nourishment,—plenty of fresh air not over-heated,—and vigorous stimulation by energetic spirits.

A county medical society aspiring to the objects quoted from the address of the president of the American medical association, must take heed to the voices of the times. It should take the initiative in each community in matters that present themselves regarding public health and the problems of sanitation. We have our canal zones and our New Orleans cesspools at our doors.

While the echoes of the recent International Congress of Tuberculosis sounding the knells of 200,000 deaths annually from a preventable disease, what is being accomplished to lessen this scourge of the white plague outside of a few large cities?

The infant mortality (Graham, Ch. Ad. Sec. Dis. of Ch.—A. M. A.), under five years of age per thousand according to the last census of the United States, is as follows: Michigan, 121.3; Connecticut, 156.8; New York, 159.8; Massachusetts, 177.8; District of Columbia, 274.5. Are these mortality figures not sufficiently appalling to stir up to activity in preventing the slaughter of the innocents? The principal cause for this high death rate is so well understood and can be so efficiently prevented, that it becomes almost criminal for a community to allow conditions to exist which contribute to such a state of affairs. The poisons given these defenseless human beings in their food, cannot be laid to criminal intent, but through the ignorance of their own mothers.

What does an average mother know about the cause of "Cholera Infantum?" The term itself fails to convey any meaning to her mind. It is a heritage of the past. Medical ignorance is deep-rooted in the minds of most people and they tenaciously cling to old ideas and superstitions. Most mothers even today believe that it is essential for a baby to have loose bowels at dentition time, or in the "second summer." If many medical terms were revised and called by their proper names much useful information would be imparted to the public. The term "cholera infantum" should be supplanted by *acute milk poisoning*. This appeals to the mind and comprehension of the most ignorant. What mother would continue to give a known poison to her offspring already made ill by a suspected food, that she knows becomes so, because of the conditions under which it is procured, marketed, and kept?

This brings me to the subject already referred to: "A New Duty of the Medical Profession: The Education of the Public in Scientific Medicine." Medical societies have a duty to perform along these lines; their members should be sources of information concerning those things which pertain to the welfare of the public in relation to the everyday conditions that menace their health. Every mother who is feeding her baby artificially on cow's milk, or other food of which it is a part, should be told the danger of doing so while the child is suffering from a putrid diarrhea. A mother never suspects for one moment that the diarrhea from which the child is sick, is caused by the germs in the very food that has heretofore perfectly nourished her baby. She has no conceptions that these germs which originally polluted the milk were some of the flora of the cow's intestinal contents, and got into the milk from particles of excreta adhering to the animal's body. She does not realise that such milk—originally pure—if allowed to remain for twenty four hours in a warm room, will contain more germs than sewage. (Chapin.)

It is our duty to instruct mothers, and the public generally, as opportunities occur with these facts, and also to advise them to patronise the dairyman who can show a clean bill of health—the man whose milk is certified that it has been produced under sanitary conditions and maintained at a temperature precluding the dangers from bacterial life. All this is possible by changed methods of the farmer, the dairyman and the dealer. The slight advance in cost consequent on the necessary care of the milk is so slight as compared to the benefits, that such a consideration should not be taken seriously into account.

Another sanitary problem confronting the public welfare is that of school hygiene. In a recent address by Dr. Thomas Darlington, Health Commissioner of the city of New York, at the eleventh annual conference of the Eastern Public Educational Associations on the "Medical Inspection of School Children in New York City," some very startling facts were adduced. "He said: "During the school year just ended 17 per cent. of the school children were excluded from attendance for varying periods of time for contagious diseases, and out of 141,908 children examined 108,329, or 76 per cent. were found to be suffering from non-contagious physical defects.

"Taking into account the time lost from school through illness, as well as acknowledging the inability of a physically defective child properly to assimilate his educational advantages, it is reasonable to assume that a large majority of these children failed of promotion because of their physical condition.

"In New York City alone (referring to the economic aspects of the question), I believe, the greater part of the $6,000,000 spent for *one year's education* of children who failed to profit by it, could have been saved by the investment of one-twentieth of that sum in proper and systematic physical examination of the children."

I quote again from the suggestive address of Dr. Burrell in this connection: "When it is recognised and brought home to the public that contagious diseases in children are to a degree unnecessary, that by proper sanitation and medical school inspection they may be in a large measure prevented, then people will demand that their little ones in public schools shall be protected against disease, which often leaves them invalided and crippled for life. A child among the better classes today, until it begins to go to school, is usually free from contagious diseases, but the moment it enters a school it is subjected to dangers from infection which it rarely escapes."

The examination of school children in districts outside the larger cities has not as yet been taken up in the state. Children residing in small towns or in the country are possessed of these same physical defects as city children,—perhaps not in so large a ratio. They have many exposures to contagious diseases in the school houses, which are generally most unhygienic, poorly cleaned and rarely ever disinfected. It is generally conceded that contagious diseases of childhood could be absolutely controlled were it not for the school house. These children should be as well cared for as those in cities. If any one will visit the crowded tenement districts of New York city and compare the school children of today with those of an older generation, he will be struck by the healthful and symmetrical faces of the former, in marked contrast to the deformed and sickly physiognomies of the latter as met on the streets. The visitor will also appreciate what this medical work among the neglected school children is accomplishing. If these measures are adding to the efficiency of school life, how much more is it going to count in fitting these same citizens for the broader fields of usefulness in the after activities of business careers where the voice, the hearing, and the eyesight are called into excessive use daily by the exigencies of our modern civilisation. The saving of life from pulmonary, throat, ear and eye diseases, and conservation of nervous systems, consequent upon neglect of these important organs in early life, is beyond computing at this time.

A word about venereal diseases. Gonorrhea, next to measles, is stated to be the most prevalent malady of civilised countries. It is estimated that from 75 per cent. to 90 per cent. of all males have been infected. It is the cause of 50 per cent. to 65 per cent. of all capital operations in public hospitals on women; and is the one factor of sterility, due to the genital infection of female infants. It is the most difficult of all infectious, contagious diseases to control in infants and foundling hospitals, asylums and day nurseries, most of whose inmates become infected; and furnishes 30 per cent. of blindness in children and over 10 per cent. of all adults in asylums. The county medical society should take more interest in the questions which are constantly presented to municipal boards of health. There is the milk problem already referred to, our potable water supply, ice supply, pure foods and drinks, as well as the control of contagious diseases.

The municipal health boards as now constituted are a reproach to our intelligence. They belong to the past, are antedated in all their methods, and inefficient in all things they undertake to perform. They are usually composed of political appointees put there for kindergarten purposes for other political offices later on. The men filling the boards are rarely ever suited by experience or fitness to judge of sanitary questions. As an example: A health board, in a neighboring municipality to where I reside, refused to concur in recommendations made by a county milk commission for regulating the principal milk supply in the county, in spite of the well-known fact, that one milk producer alone had nine out of his eighteen cows destroyed by the State Agricultural Department, because of the presence of tuberculosis. The excuse advanced, by a supervisor of the county and a member of this health board, was that the regulations would impose a hardship on a poor man with one cow, who was using it as a means of support in bringing up a family.

This selfsame board of health is referred to in the monthly bulletin of the New York State Department of Health for August, on page 211 as follows: "One board of water commissioners entered complaint against the board of health of a neighboring community, which had given permits for drains of a row of houses into a gutter, which flows directly into a tributary of the stream from which the watter supply is drawn. And the same board included among its list of twenty-five violations, the maintenance of a cesspool near a tributary by the supervisors of the county jail."

Local county health boards do nothing in sanitary matters except on complaint, founded on facts that can be sworn to if necessary, and made in writing only. This must be investigated by the local health officer. In his inspection he is often informed that the party making the complaint has probably some personal grievance and is taking this means to satisfy resentment. The health officer may be the family physician of the alleged persecuted party. His report may be biased. The action of the board depending upon the report before them defers action or compromises with one side or the other, according to their personal or political affiliations. As the Public Health law is now constituted, its defects and requirements preclude the better class of medical men accepting appointment as health officers, in many important localities.

Since I have been engaged in the preparation of this address, the September number of the monthly Bulletin of New York State Department of Health has appeared, with an editorial comment, part of which I wish to incorporate: "Some change in the cumbersome and obsolete method of procedure in local jurisdictions is much needed. An intelligent and capable health officer is often unable to get his board to act when his medical knowledge shows its necessity. Every local board of health must have been struck with the indefiniteness of the law governing its powers and duties, and its entire inability to do needed things for the protection of public health, by reason of lack of funds, and the vague provisions of the law, seemingly conferring upon them great power, but failing to specify how it can be used.

"The people of this state are, at last, fairly awake to the value and necessity of sanitary reforms, and they are demanding both from the department and from their local boards of health and health officers, effective action along many lines."

The remedy lies in making the rural boards of health as efficient as those of larger cities, and the methods and systems there in vogue could well be copied with such modification as local conditions require. The public are open to receive instruction in medical subjects.

The eagerness with which patent medicine advertisements are read by the masses well demonstrates this fact. For this reason, it is obvious why the president of the American medical association warns those who undertake the education of the public in this connection,—that we should be careful in our efforts to impart new truths that we should not convey error. Josh Billings said truly. "You better not know so much than to know so many things that ain't so."

---

A. D. Rockwell of New York has tried the effect of electricity in all its forms for the relief of neuritis without getting good results. With the use of phototherapy, he gets relief of pain dependent on congested and inflammatory conditions. He describes ten cases of neuritis treated by him by this method with marked relief, and in several with cure as a result.—*Medical Record,* November, 1907.

